# Method for spike detection from microelectrode array recordings contaminated by artifacts of simultaneous two-photon imaging

**DOI:** 10.1371/journal.pone.0221510

**Published:** 2019-08-20

**Authors:** Gábor Orbán, Domokos Meszéna, Kinga Réka Tasnády, Balázs Rózsa, István Ulbert, Gergely Márton

**Affiliations:** 1 Doctoral School on Materials Sciences and Technologies, Óbuda University, Budapest, Hungary; 2 Institute of Cognitive Neuroscience and Psychology, Research Centre for Natural Sciences, Hungarian Academy of Sciences, Budapest, Hungary; 3 Faculty of Information Technology and Bionics, Pázmány Péter Catholic University, Budapest, Hungary; 4 Institute of Experimental Medicine, Hungarian Academy of Sciences, Budapest, Hungary; Georgia State University, UNITED STATES

## Abstract

The simultaneous utilization of electrophysiological recordings and two-photon imaging allows the observation of neural activity in a high temporal and spatial resolution at the same time. The three dimensional monitoring of morphological features near the microelectrode array makes the observation more precise and complex. In vitro experiments were performed on mice neocortical slices expressing the GCaMP6 genetically encoded calcium indicator for monitoring the neural activity with two-photon microscopy around the implanted microelectrodes. A special filtering algorithm was used for data analysis to eliminate the artefacts caused by the imaging laser. Utilization of a special filtering algorithm allowed us to detect and sort single unit activities from simultaneous two-photon imaging and electrophysiological measurement.

## Introduction

Measurement methods which yield signals of neural activities with high information content, such as electrocorticography (ECoG) and intracortically implanted high density microelectrode arrays (MEAs), have vastly contributed to the progress of neuroscience and brain-computer interfacing [1–4]. MEAs are not only capable of recording the summed bioelectrical activities of neuron populations (i.e. local field potentials, LFPs), but they can also detect individual activities of neurons (i.e. single unit activities, SUAs) [5,6]. These methods had an instrumental role in the functional mapping of the brain [7] and they are still the ultimate solution when high spatial and temporal resolution are required [4,8,9]. However, the spatial range of the SUA detection capability of the implanted sensors is limited to the immediate surroundings the electrode sites, i.e. hundred micron wide volumes [10]. Furthermore, the long term use of implanted MEAs is corrupted by the degradation of their performance over weeks or months, let alone years [11–13]. The underlying causes range from material failures [14,15] to the deteriorative effects of the immune response to the implants [16–18].

In the last few decades, various optical imaging methods became widely used in neuroscience, which can render wide brain regions observable with high spatial resolution [19–23]. Furthermore, the application of two-photon microscopy with fluorescent calcium indicators makes the monitoring of neural activity (e.g. action potentials of individual cells) possible [24–27]. The focal length of fluorescence microscope objectives can exceed 12 mm [28], which allows the implantation of depth MEAs into the optical cranial window [29].

Simultaneous application of depth MEAs for extracellular electrophysiology and two-photon imaging could allow neuroscientists to observe activities of individual neurons with good spatial and temporal resolution at the same time, thus the more precise and complex pieces of information could be obtained from neural activity [30]. The extension of high density intracortical recordings with simultaneous two-photon microscopy would enable three dimensional optical monitoring of the structural features of the cells located close to the electrode.

Nonetheless, the co-localized and simultaneous application of two-photon imaging and electrophysiological measurement by MEAs remains challenging, partly because of the photoelectric artefacts on the electrophysiological recordings caused by the imaging laser [31]. The artefacts generally appear as huge sawtooth-like waves. The main frequency of such waves correspond to the imaging frame rate of the applied two-photon laser. The frame rate of the imaging is indeterminate, moreover, the sharp shape of the waves and other effects introduce various harmonics other than the main frequency, thus elimination of the photoelectric artefacts requires more subtle methods than applying e.g. a notch filter. Comb filters have already been successfully used for decreasing stimulus artefacts [32] and 50 Hz low frequency noise [33] from electrophysiological signals, while adaptive filters are utilized e.g. in brain-computer interface development [34,35], in simultaneous measurements of real-time magnetic resonance imaging and electrocardiogram recordings [36], in fetal electrocardiogram analysis [37], etc.

In this study we present a method wherein the utilization of a custom-set comb filter based algorithm allowed us to detect and sort SUAs from extracellular multi-channel recordings while the measuring electrode array and the surrounding tissue was monitored with two-photon imaging. The method was validated by applying the filter on recording sections when the imaging laser was not in use and checking whether the introduced laser artefact affects SUA detection and clustering.

## Materials and methods

### Preparation of in vitro experiments

In vitro experiments were performed on mice expressing the GCaMP6 genetically encoded calcium indicator for the monitoring of neural activity around the MEA [38,39].

A total of three GCaMP mice had been anesthetized with a ketamine-xylazine solution and prepared for operation as described elsewhere [40]. Animals for acute tests were kept and handled in accordance with the European Council Directive of 24 November 1986 (86/609/EEC), the Hungarian Animal Act, 1998 and the Animal Care Regulations of the Research Centre for Natural Sciences of the Hungarian Academy of Sciences (RCNS-HAS). The study was approved by the Institutional Animal Care and Use Committee of the Research Centre for Natural Sciences of the Hungarian Academy of Sciences (members: Dr. István Ulbert, Dr. József Topál and Péter Kottra) and the National Food Chain Safety Office of Hungary (PEI/001/695-9/2015). Animals had unlimited access to food and water, when they were awake. Each mouse was kept in a 39 cm long, 22 cm wide, 18 cm high cage. They were under deep anesthesia during surgery as well as at the time of sacrifice. Efforts were made to minimize animal suffering and to reduce the number of animals used.

Cortical and hippocampal slices were prepared from the mice brains. The brains were immediately removed and dipped into cold (2–3°C), oxygenated (95% O_2_, 5% CO_2_) cutting solution. The cutting solution contained the following composition (in mM): 250 Sucrose, 26 NaHCO_3_, 10 D-Glucose, 1 KCl, 1 CaCl_2_ and 10 MgCl_2_. 500 μm-thick horizontal slices were cut by a vibratome (VT1200s; Leica, Nussloch, Germany) from both hemispheres. Slices were kept in a standard artificial cerebrospinal fluid (aCSF) solution at room temperature (20–22°C) for at least 1 hour before use. The recordings were performed at 32–34°C with a standard recording aCSF containing (in mM): 124 NaCl, 26 NaHCO_3_, 10 D-Glucose, 4 KCl, 2 CaCl_2_ and 2 MgCl_2_. In the recording chamber, a dual-perfusion system was used by perfusing both the top and the bottom surfaces of the slices with relatively high perfusion speed (>10 ml/min) to provide better oxygenation, similar to in vivo conditions [41].

### Two-photon imaging

The three dimensional observation of morphology was performed with two-photon microscope (Femtonics Ltd., Budapest, Hungary). The two-photon imaging not only let us monitor the neural activity near the applied MEA because of the genetically encoded calcium indicator expressing GCaMP6 cells, but it also made the observation of imaging laser generated artefacts possible. For the optical imaging the prepared slices were placed into an in vitro measuring chamber. The chamber ensured the aCSF supplement and circulation for keeping the neural tissue alive until the end of the measurement and it stabilized the slice mechanically with a holder mesh. The top part of the chamber is concave-shaped to hold the aCSF for the liquid immersion objective of the two-photon microscope which was used during the experiments. The applied laser had a wavelength of 940 nm and worked in resonant mode.

The setup was not only able to function in two-photon imaging mode but it also did work in camera mode which allowed us to follow the track of the inserted MEA before and during the insertion because of the built-in CCD camera. With the brain slice in place, the bioelectrical activity was monitored in two-photon mode and the electrophysiological measurement setup was assembled.

### Electrophysiological measurement

**Fig 1:**
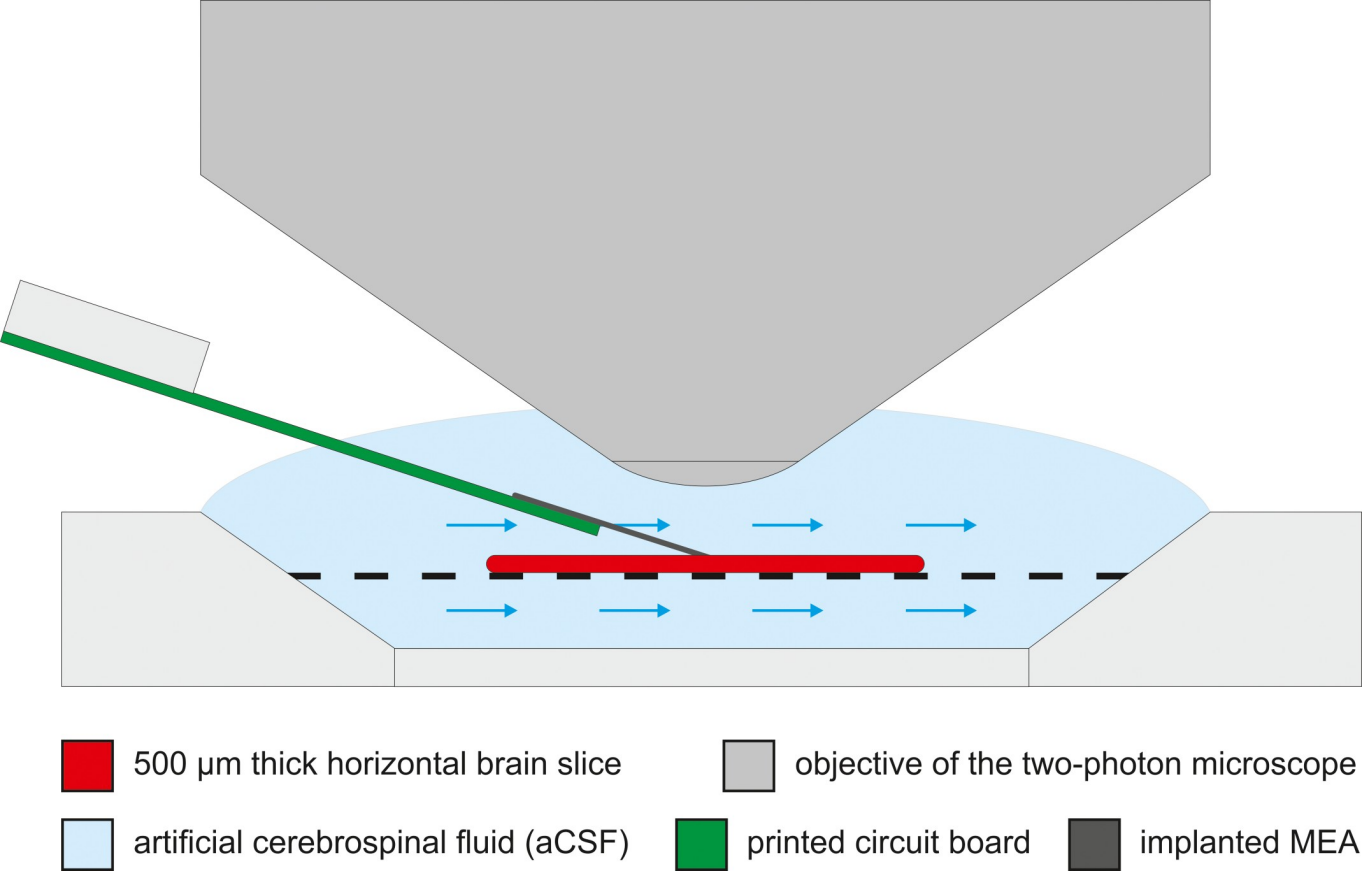
Schematic of the assembled measuring system. In the middle of the in vitro two-photon measuring chamber the brain slice is placed on a holder mesh. The chamber provides the aCSF circulation near the neural tissue to keep it bioelectrically active. Under the fluid immersed two-photon objective the applied MEA was inserted into the tissue.

**Fig 2:**
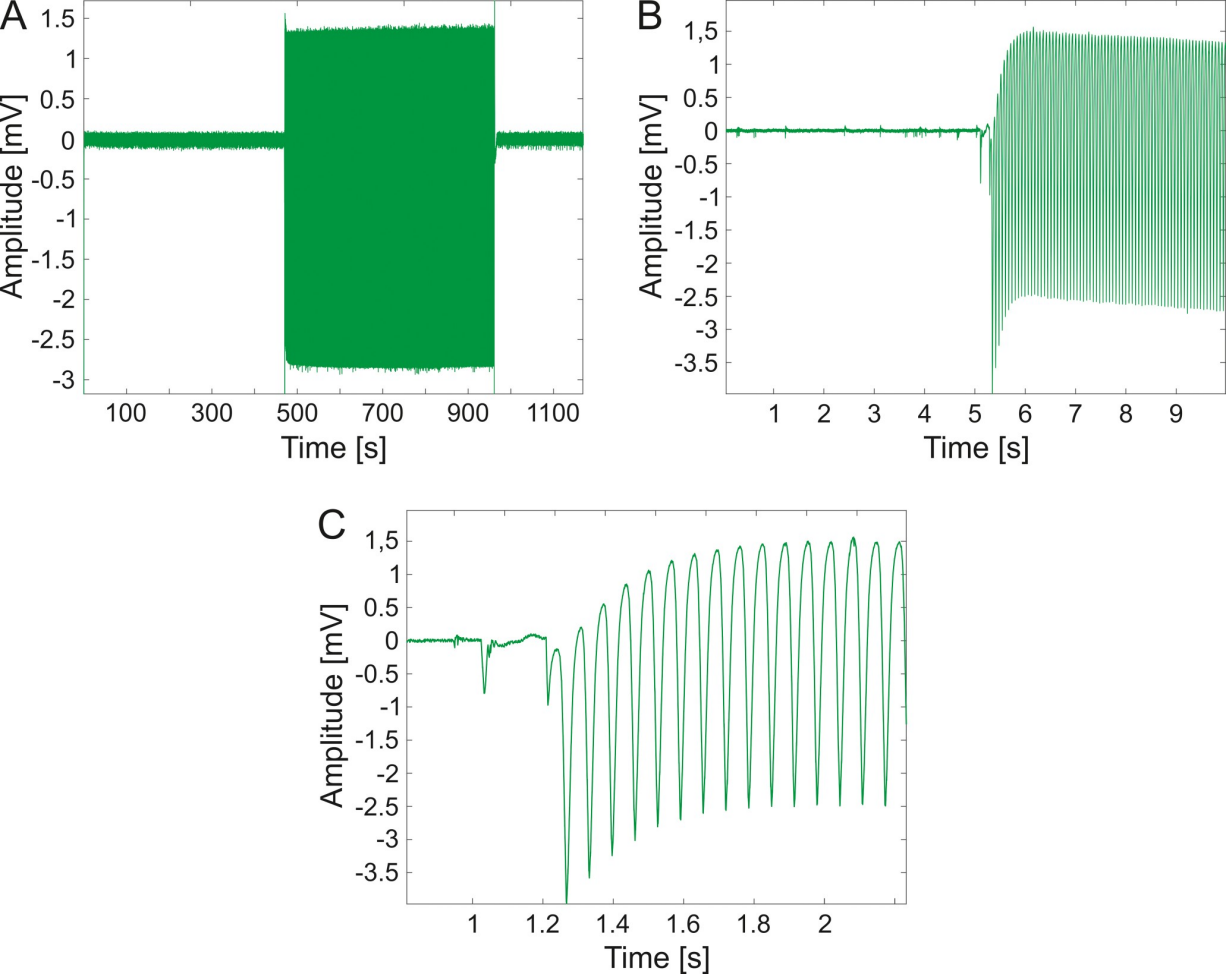
Representative sample of the imaging laser impact on the electrophysiological recordings. Between the first and the last parts of the measurement, which were recorded without two-photon imaging, photoelectric artefacts of the two-photon imaging laser are observable (A). The recorded data at the moment when the imaging laser was switched on (B, C).

The electrophysiological observation of the bioelectrical activity of the examined brain slices were carried out using an Intan RHD—2000 amplifier system (Intan Technologies LLC., Los Angeles, CA, USA) connected to a computer via USB 2.0 with a sampling frequency of 20 kHz. The reference electrode was an Ag/AgCl needle located beneath the tested brain slice. A MEA with 16 shanks (1 electrode/shank) (A16x1-2mm-50-177-A16, NeuroNexus, Ann Arbor, MI. USA) was employed as a working electrode. The MEA was attached to one of the automated electrode holder of the two-photon setup in such a manner that the longitudinal axes of the shanks included an angle of 30 degrees relative to the surface of the brain slice. The implantation was performed under CCD camera control. After the MEA had reached its final place in the tissue, the two-photon setup was switched from camera mode to two-photon mode and the electrode sites were located. The schematic of the assembled experiments is shown in Fig 1. Following this, the focal plane was stabilized and the imaging laser was turned off. The electrophysiological measurement was started without the laser in order to provide reference recordings. After 8 minutes of such laser-free recordings, the two-photon imaging was initiated and the imaging laser introduced artefacts. Another 8 minutes of laser-noised recordings were hence obtained. The third part of each measurement was performed without the two-photon imaging again, in order to obtain further control data sessions, this time after the laser effect. During the second part of the measurements, the laser generated artefacts which exceeded the amplitude of SUAs by at least an order of magnitude (Fig 2).

### Data analysis

**Fig 3:**
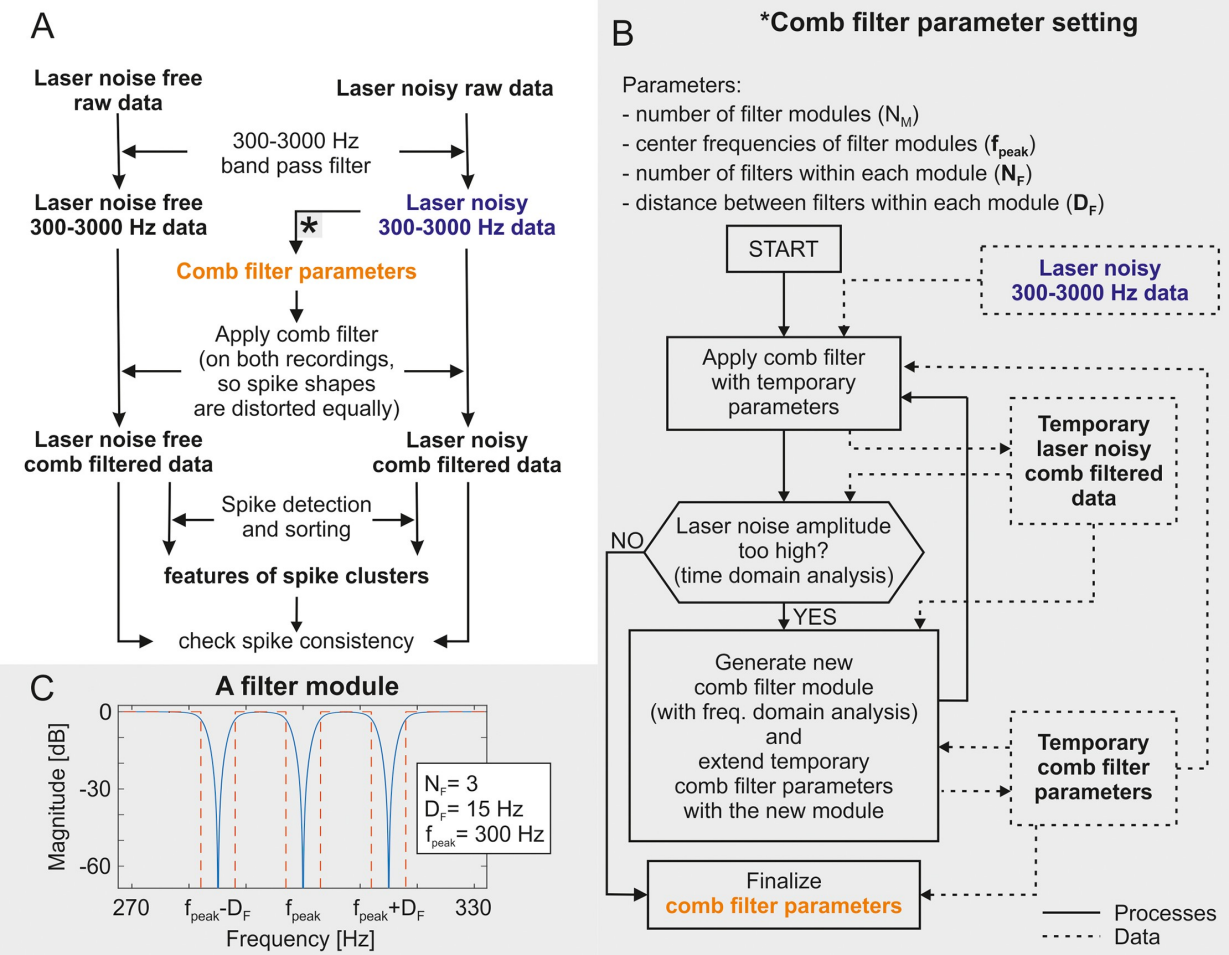
Filtering and analyzing steps. The performed filtering and analyzing steps in order to identify the spike clusters and check the spike consistency between the two-photon imaging laser noise free and the laser noisy data (A), the parameter setting algorithm of the applied custom-set comb filter (B) and the result of the parameter setting of a representative filter module (C).

MATLAB 2017a (MathWorks Inc., Natick, MA, USA) was used for off-line signal visualization, filtering and analysis. Fig 3 summarizes the steps that had been performed in order to accomplish our ultimate goal, i.e. the identification of spike clusters in the data containing two-photon laser noise.

**Fig 4:**
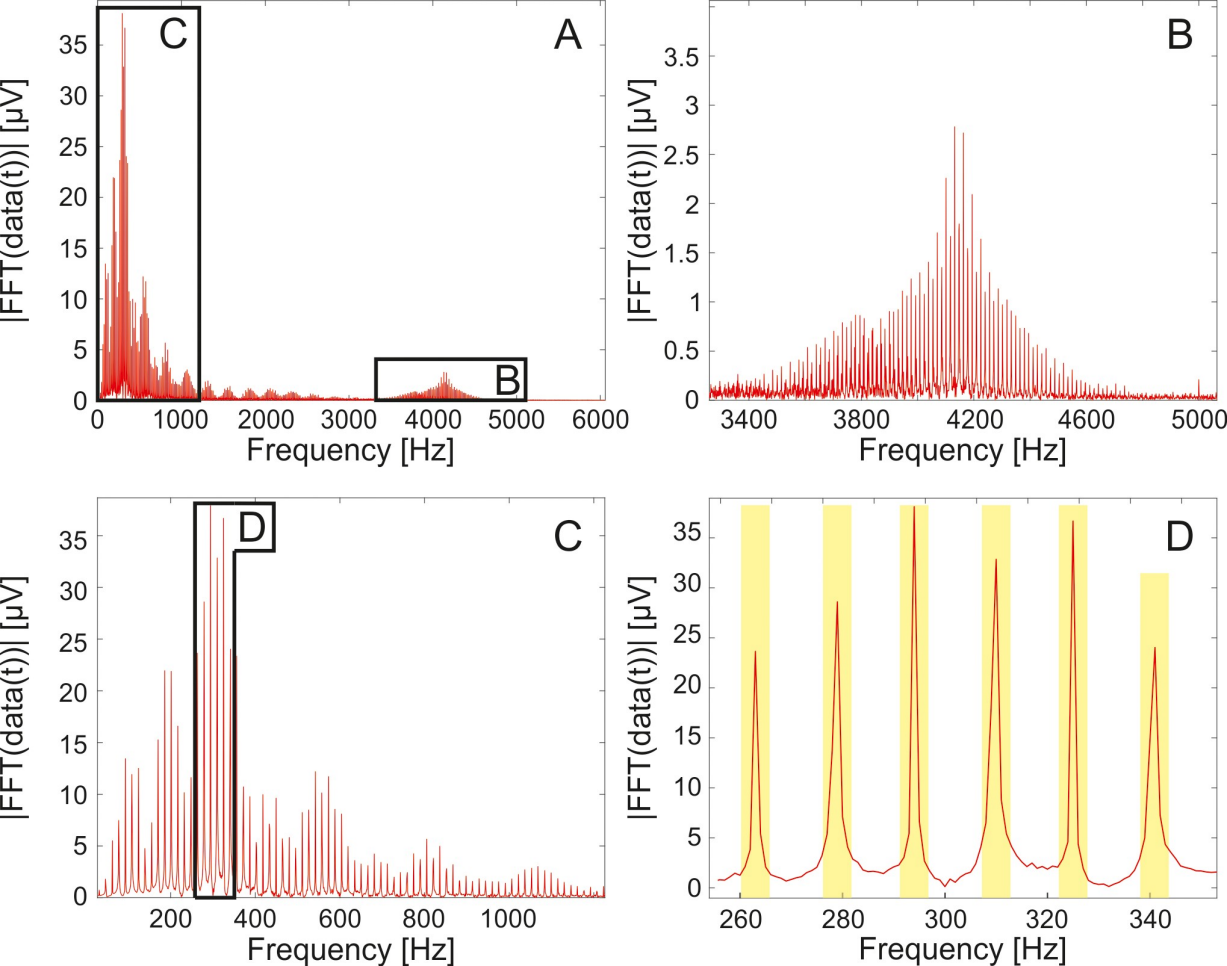
The absolute value of the frequency spectrum of the electrophysiological recordings. The fast Fourier transform analysis of the imaging laser generated noise in the electrophysiological recorded data (A). Harmonics below 1200 Hz (C) and at higher frequencies (B) of the laser generated periodical artefacts appeared with high magnitudes. The overlap of the harmonics is observable (B). A part of the rejected frequencies by the custom-set comb filter is shown in yellow (D).

All of the applied band-stop IIR filters were created with passband ripples of 0.4. Since the IIR filters delay some frequency components more the others, they distort the input signals with frequency dependent phase shift. Thus they were applied with the ‘filtfilt’ Matlab function that compensated the delays introduced by such filters, and thus corrected for filter distortion. The recorded signals were initially filtered with a second order band-pass filter between 300 Hz and 3000 Hz, which is a commonly used method for highlighting and detecting SUAs [42], but not adequate for eliminating the photoelectric artefacts. Following this, Fast Fourier transform (FFT) was applied on the electrophysiological recordings. Comparing the frequency spectra of the first (laser off) part of each measurement to their second part (laser on), it was evident that the imaging laser gave rise to a population of high peaks in the frequency domain. These peaks were located periodically, with a distance of 15.5 Hz between the neighboring ones. This frequency value corresponds to the imaging frame rate of the applied two-photon laser (Fig 4). Considering this nature of the artefacts, it is a straightforward idea to utilize of a comb filter algorithm to eliminate the noise of the imaging laser. Fig 3 shows the whole data evaluation process for spike sorting (Fig 3A), including the construction of the laser noise reduction filters (Fig 3B). Such a comb filter had to be constructed individually for every recording channel because of the different laser noise characteristics on the channels. Each custom-set comb filter was built from filter modules, a representative filter module is shown in Fig 3C. The modules contain band-stop filters fitted to a certain amount of peaks in the frequency domain.

The parameter setting algorithm of the comb filter is shown in Fig 3B. These parameters were the number of filter modules (N_M_), a vector containing the center frequencies of the filter modules (**f**_**peak**_), the numbers of the applied band-stop filters within each module (**N**_**F**_) and the distances between the center frequencies of the applied band-stop filters within each module (**D**_**F**_). The parameter setting algorithm utilized the 300–3000 Hz filtered laser noisy data in a cyclic manner, during each cycle, a new filter module is added to the comb filter. The first step in the cycle was the generation of a temporary laser noise filtered data by the application of the temporary comb filter, i.e. the comb filter generated in the previous cycle on the 300–3000 Hz filtered laser noisy data (in the first cycle the number of filter modules is 0, so this step left the data unchanged). The second step was deciding whether the temporary filter was sufficient. This was performed by time domain analysis on the temporary laser noisy filtered data. If the amplitude of the periodic laser noise had been reduced below 40 μV, then the temporary filter parameters became the finalized comb filter parameters. Otherwise, the last step in the cycle followed, which was the generation of a new filter module. This was performed based on the frequency domain analysis of the temporary laser noisy comb filtered data (which is equivalent to the 300–3000 Hz filtered laser noisy data in the first step). After applying the FFT on this data, the algorithm found the highest peak in the frequency domain. This frequency became the center frequency (f_peak_) of the new filter module. The neighboring peaks were located at the frequencies of f_peak_ ± n∙D_F_ (D_F_ was found to be 15.5 Hz). The values at the neighboring peaks were compared to the highest detected peak to define the number of the applied filters (N_F_) within the new module. N_F_ of the filter module was defined so that the band-stop filters of the comb filter would cover all the neighboring peaks which exceeded in height the 15% of the highest peak (i.e. the one at the center frequency). Every band-stop filter element of the new comb filter module was defined with cutoff frequencies at below 3 Hz and above 3 Hz from the frequency value of each peak. Thus the central rejected frequencies of the comb filter were adjusted to the frequencies of the laser noise peaks and each section of the comb filter had a 6 Hz wide rejected band, as shown in Fig 3C and in Fig 4D. The temporary comb filter was extended with the thus obtained new module and the cycle restarted. This process was repeated until the time domain analysis gave positive result, i.e. the amplitude of the laser noise peaks in the time domain became lower than 40 μV, in which case the summarized comb filter parameters were accepted.

As shown in Fig 3A, the thus constructed comb filters were applied on both the laser noise free and the laser noisy 300–3000 Hz filtered data in order to equally distort the SUA (“spike”) waveforms in both cases. Later on, this allowed us to match the features of different spike clusters in the laser free and laser noisy measurements. Since the imaging laser generated artefacts were nonuniform along the electrodes, recordings from different electrodes required filters with custom-set parameters.

**Fig 5:**
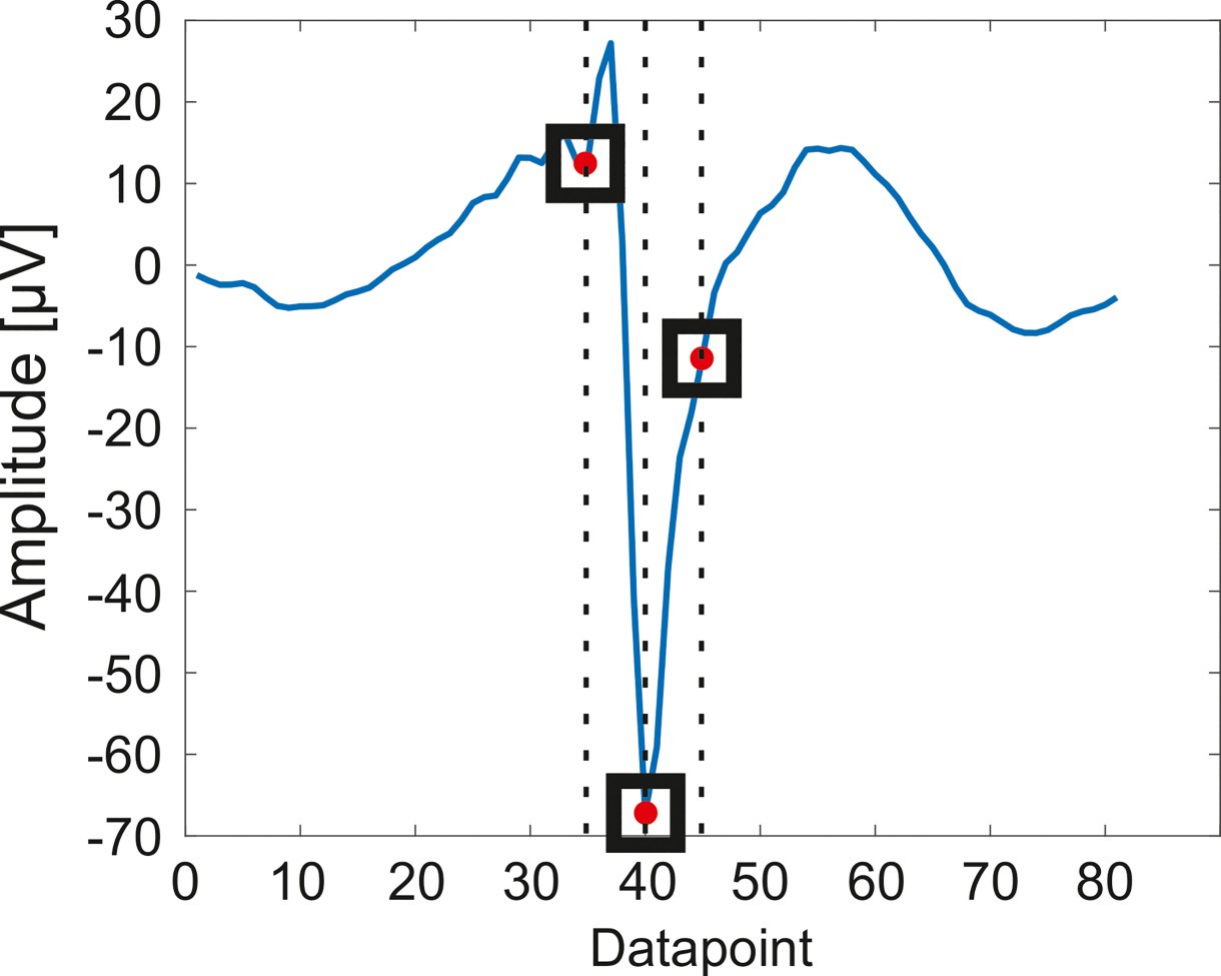
The applied principal component selection. Each potential spike was defined with their three principal component before spike sorting: the location of the minimum amplitude value of the spike, and the fifth datapoints before and after the peaks.

We investigated whether the comb filter prevents us from SUA (“spike”) detection and sorting. Spike detection was performed by simple thresholding. Three features of each potential spikes were defined for spike sorting, which were the location of the minimum amplitude value of the spike, and the values at 250 microseconds (i.e. five datapoints) before and after the peaks (Fig 5). The clusters were manually accepted or discarded based on spike waveforms and autocorrelograms. This feature extraction method was preferred rather than principal component analysis (PCA), because the thus defined features could provide more robust information about spike waveform consistency (spike stability). In terms of the laser noise free part of the experiments, we performed a comparison of the feature-based and the PCA methods on the band-pass filtered data to verify the results of the feature extraction based method which was used for testing the spike stability too. The spike stability was verified as follows. First, the averages and the standard errors of the means of each feature were calculated in every minute of the recordings. These values were compared to each other during the whole measurement to verify the impact of the imaging laser and the applied filters to the shape of the thus sorted spikes. Furthermore, the number of spikes were counted in every minute of the recordings for each clusters. This method showed whether the artefacts caused by the imaging laser gave rise to false positive SUA detections.

## Results and discussion

As shown previously, simultaneous two-photon imaging and electrophysiological measurements with MEMS microelectrode arrays at the same location is compromised by the formation of photoelectric artefacts in the electrophysiological signals. Regarding our experiments when electrodes were located within the two-photon imaging window, the imaging laser was able to create such artefacts with amplitudes of typically 50 times greater than the amplitude of the largest single unit activities. Moreover, the complicated spectrum of the photoelectric noise prevents the filtering of the artefact via simple filters.

**Fig 6:**
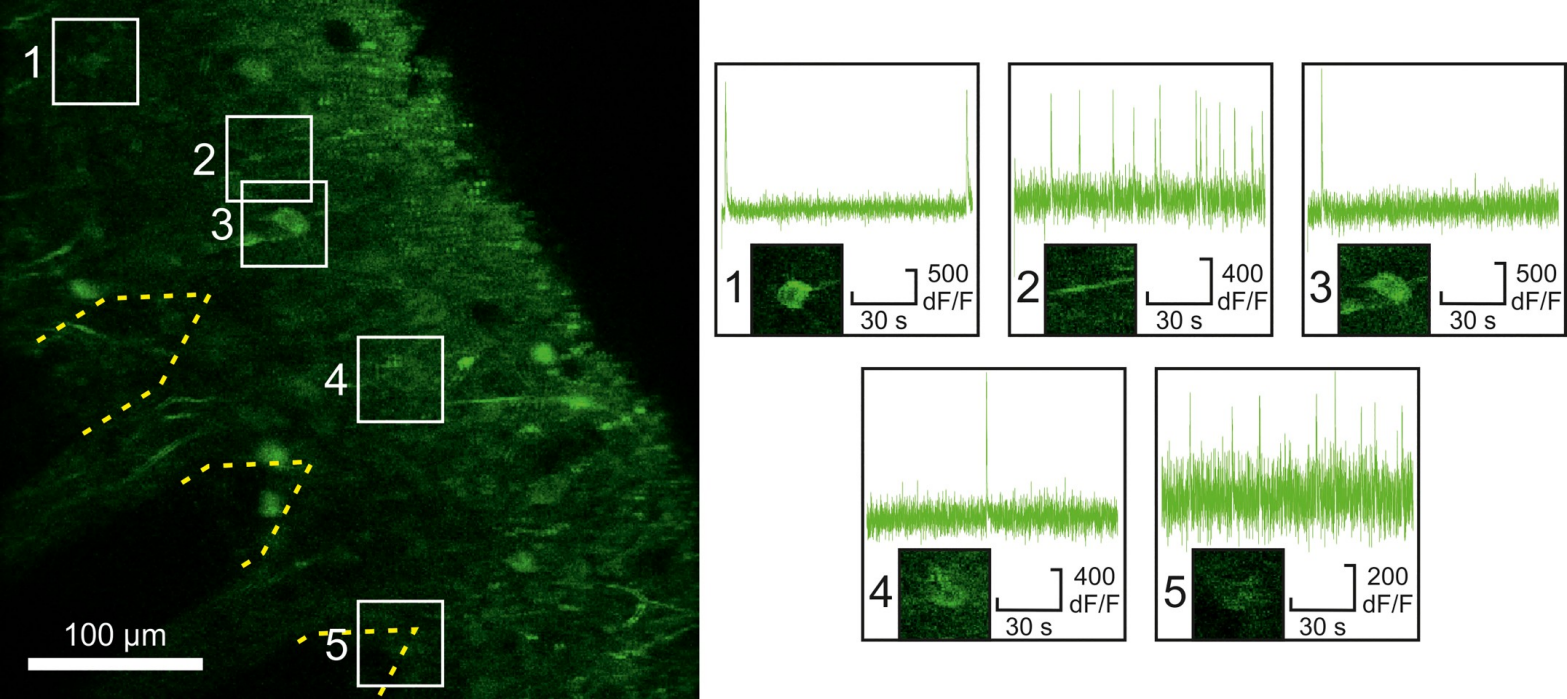
Two-photon calcium imaging. The imaging reveals activities of neuron somas (subfigures 1, 3, 4, 5) and dendrites (subfigure 2) in the vicinity of the microelectrode array.

Our following results suggest that the utilization of a comb filter-based algorithm can enable researchers to detect and sort single unit activities even if the tissue surrounding the microelectrode array is observed with two-photon microscopy. Fig 6 illustrates the observed area and suggests that the above described two-photon microscope setup and settings were suitable for detecting activities of neuron somas and dendrites via calcium imaging.

**Fig 7:**
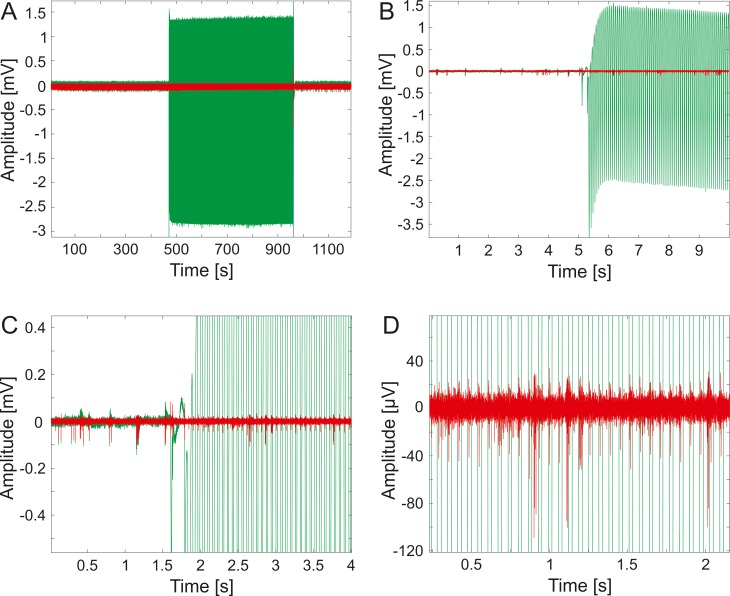
Representative sample of the results of the applied filtering algorithm. The subfigures show the same data as Fig 2 does, prior to filtering (green) and after applying the filtering algorithm (red).

The filters influenced frequency spectrum of the electrophysiological recordings is shown in S1 Fig, where subfigure A shows the absolute value of the frequency spectrum of the unfiltered signal, subfigure B shows the absolute value of the frequency spectrum of the band-pass filtered signal while subfigure C shows the absolute value of the frequency spectrum of the band-pass and noise filtered signal. Comparing the subfigures, it can be observed that after both of the filtering processes the frequency component of the noise became two orders of magnitude lower. Fig 7 shows neural signal samples obtained from an electrode illuminated with direct laser light before (green) and after (red) the application of the filter. It is evident that small amplitude spike-like artefacts are still present on the filtered signal and these spike-like artefacts are synchronized with the period of the laser noise. Fortunately, however, we can also observe that major single unit activity amplitudes exceed the amplitude of these artefacts. The filter was also applied on the signal sections which were recorded when the imaging laser was off so those sections can serve as proper references for single unit activity detection. Moreover, with further developments, the artefact spikes can probably be eliminated with an algorithm which takes into account the synchrony of the artefacts and the laser noise. A limitation of this proposed method is that when a single unit activity coincides with a spike artefact, it is probably also eliminated. However, comparing the width and the density of the laser generated artefacts in time range, this limitation should only affect approximately 8.5% of the signal.

**Fig 8:**
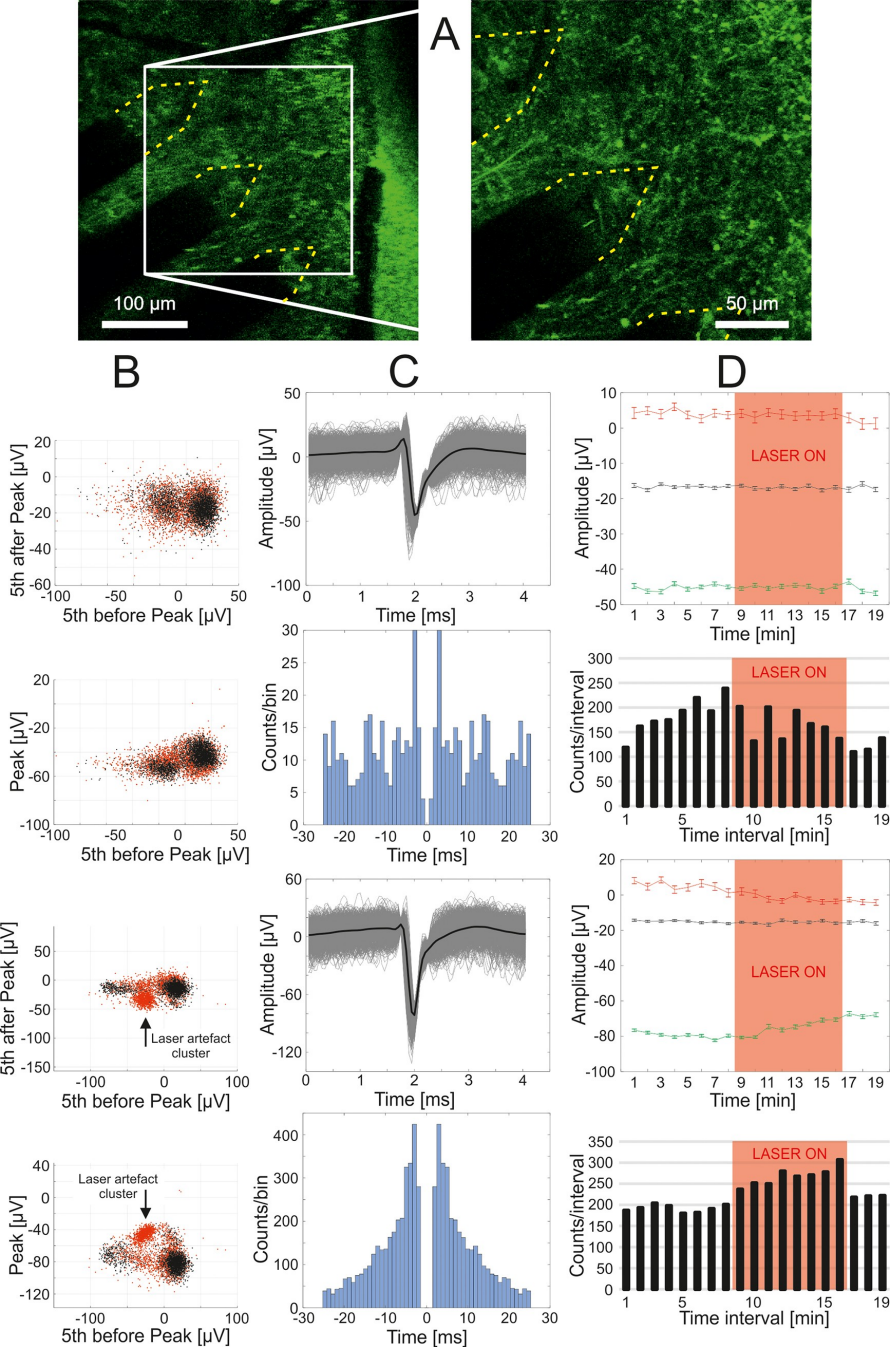
Representative results of the simultaneous measurement of electrophysiological recordings and two-photon imaging. The examined electrodes of the applied MEA were in the two-photon imaging window (A). Two examples of sorted spikes recorded by the electrodes shown in part A (B-D). Potential spikes were sorted using three features obtained from the comb-filtered signals (B). The obtained spike waveforms, their averages (C, top) and the autocorrelograms of the thus sorted spikes (C, bottom). The verification of spike stability during laser noise free and the simultaneous two-photon measurements (containing laser noise) is presented in part D. Changes in the shapes of the detected spikes were observed by comparing the averages and standard errors of spike features during the measurements (D, top). The number of the sorted spikes per minute (D, bottom).

Fig 8A shows the tissue region observed with two-photon microscopy, containing the recording electrode sites. The result of the feature extraction for this representative case is shown in Fig 8B, where the potential spikes are shown in black (detected during the laser off condition) and red (detected during the laser on condition). The obtained spike waveforms, their averages and autocorrelograms in Fig 8C. The differences between the laser on and off conditions are shown in S3 Fig (spike waveforms and their averages) and S4 Fig (autocorrelograms). The results of the comparison of the feature extraction and the PCA based methods for spike sorting is shown in S5 Fig. To verify the applied filtering algorithm, the consistency of the sorted spike waveforms (spike stability) was visualized. Results of the average of spike features within each minute of the recordings suggest that the laser noise does not corrupt the thus obtained spike waveforms (Fig 8D, top). Furthermore, the number of the spikes in each minute of the measurement suggest that the laser noise does not introduce artefact spikes into the clusters (Fig 8D, bottom). A further result is presented in supplementary S2 Fig, which shows a histogram of the occurrence of each spike within the laser noise period. This result may be caused by modulations of the cells firing rates due to the laser light, as suggested by Kozai et al [43].

We agree with neuroscientists claiming that simultaneous application of two-photon imaging and implanted MEAs would be beneficial for obtaining more complex information about the activity, connectivity and function of brain cells [31,44,45]. One of the major challenges of the simultaneous utilization of these state of the art methods is caused by the photoelectric artifacts on the electrophysiological signals caused by the imaging laser. This challenge was partly overcome previously with various data filtering algorithms [46]. The herein presented filtering method alleviates this hindrance further by offering means for researchers to detect and sort SUAs from recordings infected by the laser noise of a two-photon microscope. However, the methods have still limitations. A “clean”, laser noise free recording is suggested to be recorded before and after the actual simultaneous recording in order to verify the validity of the obtained spike features. Furthermore, the applied comb filter distorts spike waveforms more than the more commonly utilized band-stop filters (with cutoff frequencies at e.g. 300 Hz and 3000 Hz). The presented method can be further developed by the application of an automated algorithm which determines the range of the comb filter in the frequency band, and by a more complex software which takes into account the periodicity of the laser noise for spike detection. Some efforts were made for automating the process, i.e. to solve the parameter setting step automatically, but for a sufficiently robust algorithm more work needs to be done on this matter.

## Conclusion

In this paper we presented a method for recording extracellular signals with depth microelectrode arrays and two-photon images within the same tissue region, simultaneously, in a such manner that even single unit activities can be obtained from the electrophysiological recordings. To our knowledge, this is the first time that the possibility of obtaining such data has been presented. The applied filtering algorithm was capable of eliminating the majority of the periodic photoelectric artefacts generated by the imaging laser in order to allow us to perform single unit activity detection and sorting. The method might allow researchers to employ two-photon microscopy in order to reveal crucial properties of high density extracellular neurophysiology and vice versa. The application of simultaneous, multimodal measurements might give rise to novel findings in neuroscience and effective brain-computer interfaces.

## Supporting information

S1 Fig The effect of the filters on the frequency spectrum of the electrophysiological recordings. Subfigure A shows the absolute value of the frequency spectrum of the unfiltered signal, subfigure B shows the absolute value of the frequency spectrum of the band-pass filtered signal. Subfigure C shows the absolute value of the frequency spectrum of the band-pass and noise filtered signal.
(TIF)

S2 Fig The number of the detected neuronal spikes compared to the start of the laser imaging frame acquisition cycle. Subfigures A and B show the occurrence of two sorted spikes presented in Fig 8. It revealed that the firing of the detected cells is modulated with the laser light, as also shown by Kozai et al. [43].
(TIF)

S3 Fig The detected spike traces and their averages during the laser on and off conditions for two clustered units. The related A-B and C-D subfigures present the first laser off (A and C) and the laser on (B and D) conditions.
(TIF)

S4 Fig The difference between the autocorrelograms during the laser on and off conditions for two clustered units. The related A-B and C-D subfigures present the first laser off (A and C) and the laser on (B and D) conditions.
(TIF)

S5 Fig The result of the comparison of two spike sorting methods. The Principal Component Analysis of the laser noise free, band-pass filtered data (A) in the two cases of the presented single unit activities of Fig 8. The detected and sorted SUA waveforms with their averages based on the PCA (B, top) and based on the feature extraction methods (C, top). The interspike interval (ISI) violators based on the PCA (B, bottom) and based on the feature extraction methods (C, bottom).
(TIF)
