# Correction: Method for spike detection from microelectrode array recordings contaminated by artifacts of simultaneous two-photon imaging

**DOI:** 10.1371/journal.pone.0224590

**Published:** 2019-10-24

**Authors:** Gábor Orbán, Domokos Meszéna, Kinga Réka Tasnády, Balázs Rózsa, István Ulbert, Gergely Márton

The affiliation for the fifth author is incomplete. István Ulbert is also affiliated with #2: Institute of Cognitive Neuroscience and Psychology, Research Centre for Natural Sciences, Hungarian Academy of Sciences, Budapest, Hungary

## References

[1] Orbán G, Meszéna D, Tasnády KR, Rózsa B, Ulbert I, Márton G (2019) Method for spike detection from microelectrode array recordings contaminated by artifacts of simultaneous two-photon imaging. PLoS ONE 14(8): e0221510 10.1371/journal.pone.0221510 31430357 PMC6701834

