# Supplementary figures and images for: Method for spike detection from microelectrode array recordings contaminated by artifacts of simultaneous two-photon imaging
Source: PLoS One. 2019 Aug 20;14(8):e0221510. doi: 10.1371/journal.pone.0221510 (PMC6701834; doi:10.1371/journal.pone.0221510)

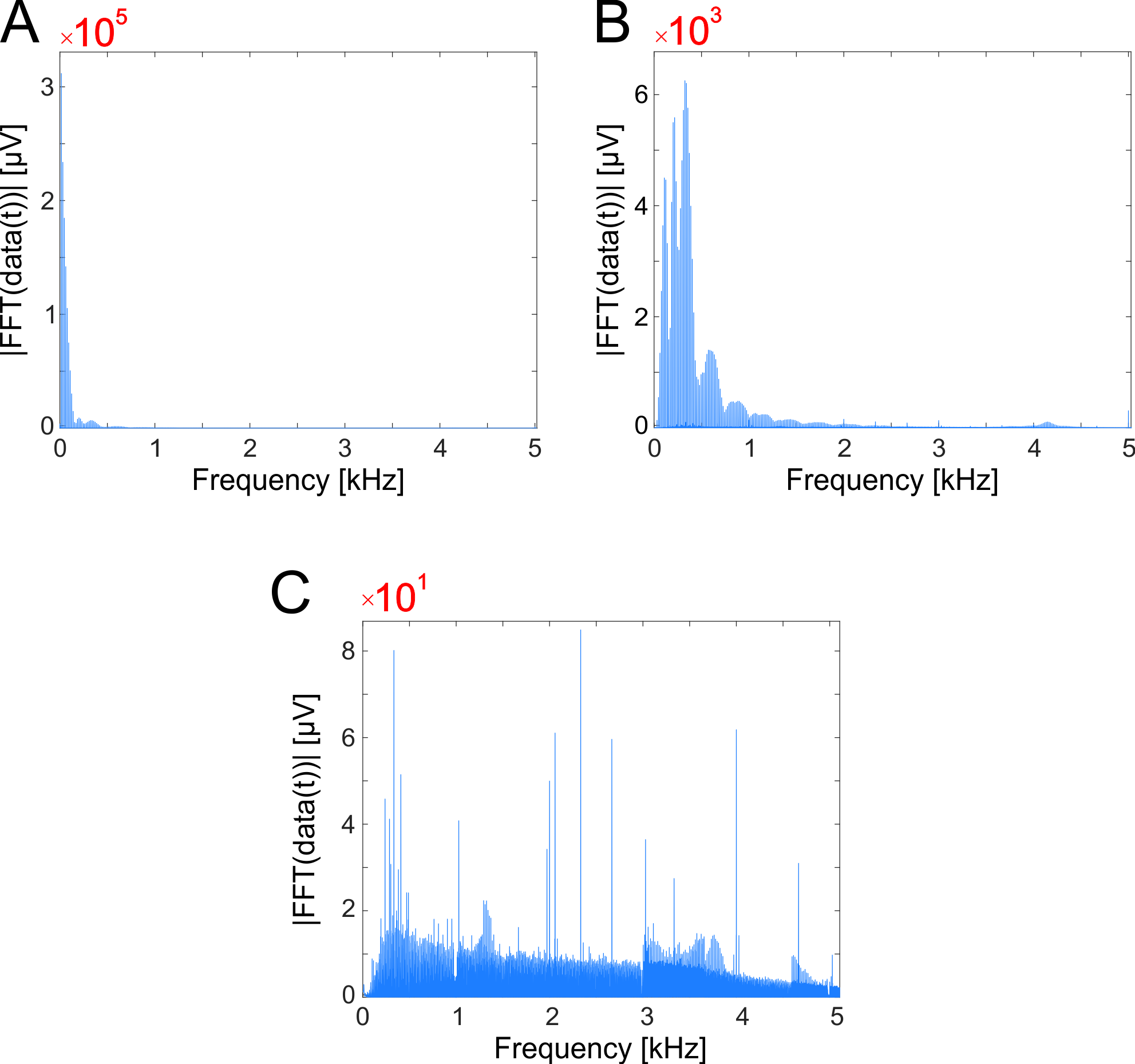

Supplement: S1 Fig — Subfigure A shows the absolute value of the frequency spectrum of the unfiltered signal, subfigure B shows the absolute value of the frequency spectrum of the band-pass filtered signal. Subfigure C shows the absolute value of the frequency spectrum of the band-pass and noise filtered signal. (TIF) [file pone.0221510.s001.tif]

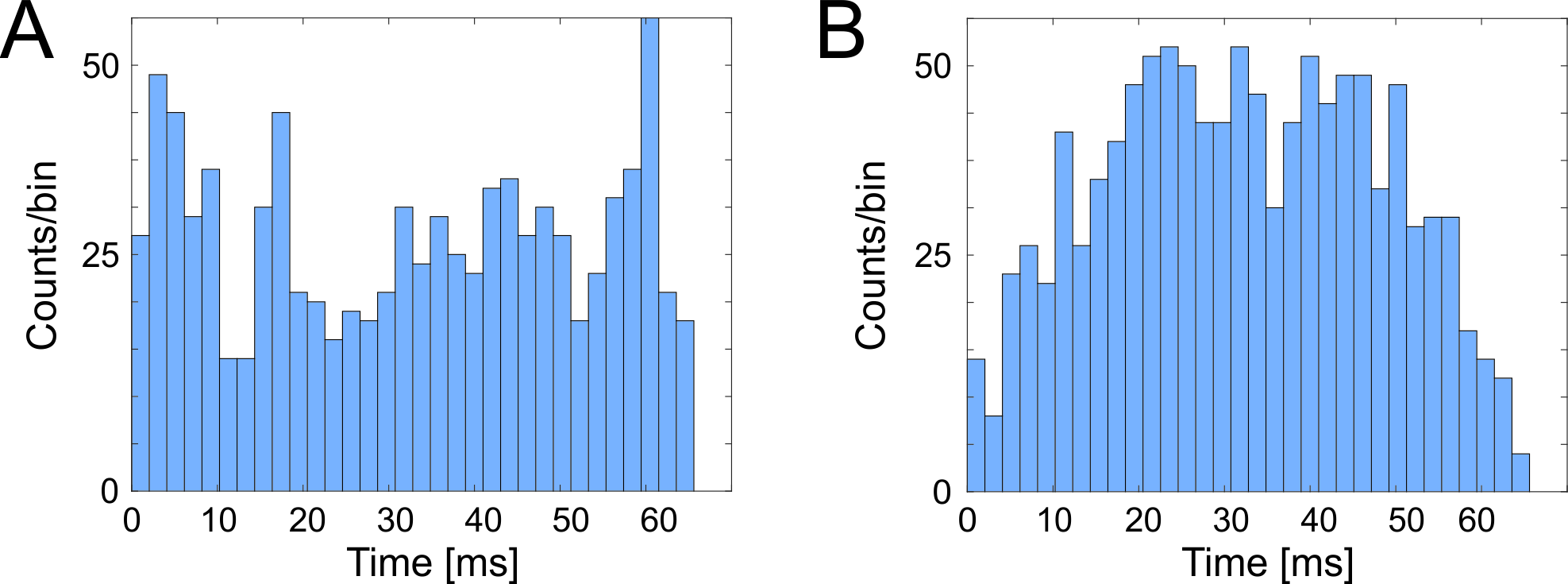

Supplement: S2 Fig — Subfigures A and B show the occurrence of two sorted spikes presented in Fig 8. It revealed that the firing of the detected cells is modulated with the laser light, as also shown by Kozai et al. [43]. (TIF) [file pone.0221510.s002.tif]

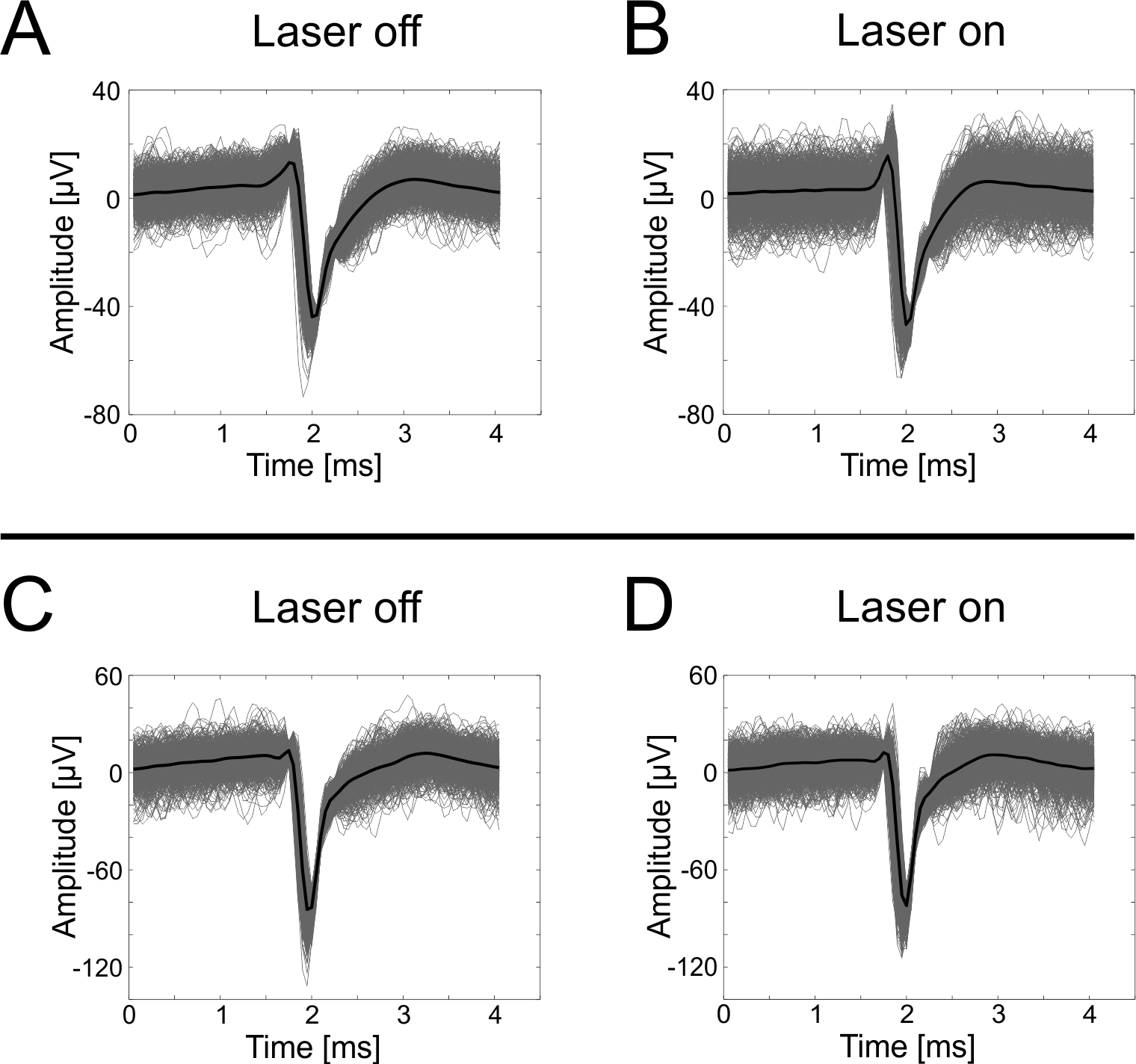

Supplement: S3 Fig — The related A-B and C-D subfigures present the first laser off (A and C) and the laser on (B and D) conditions. (TIF) [file pone.0221510.s003.tif]

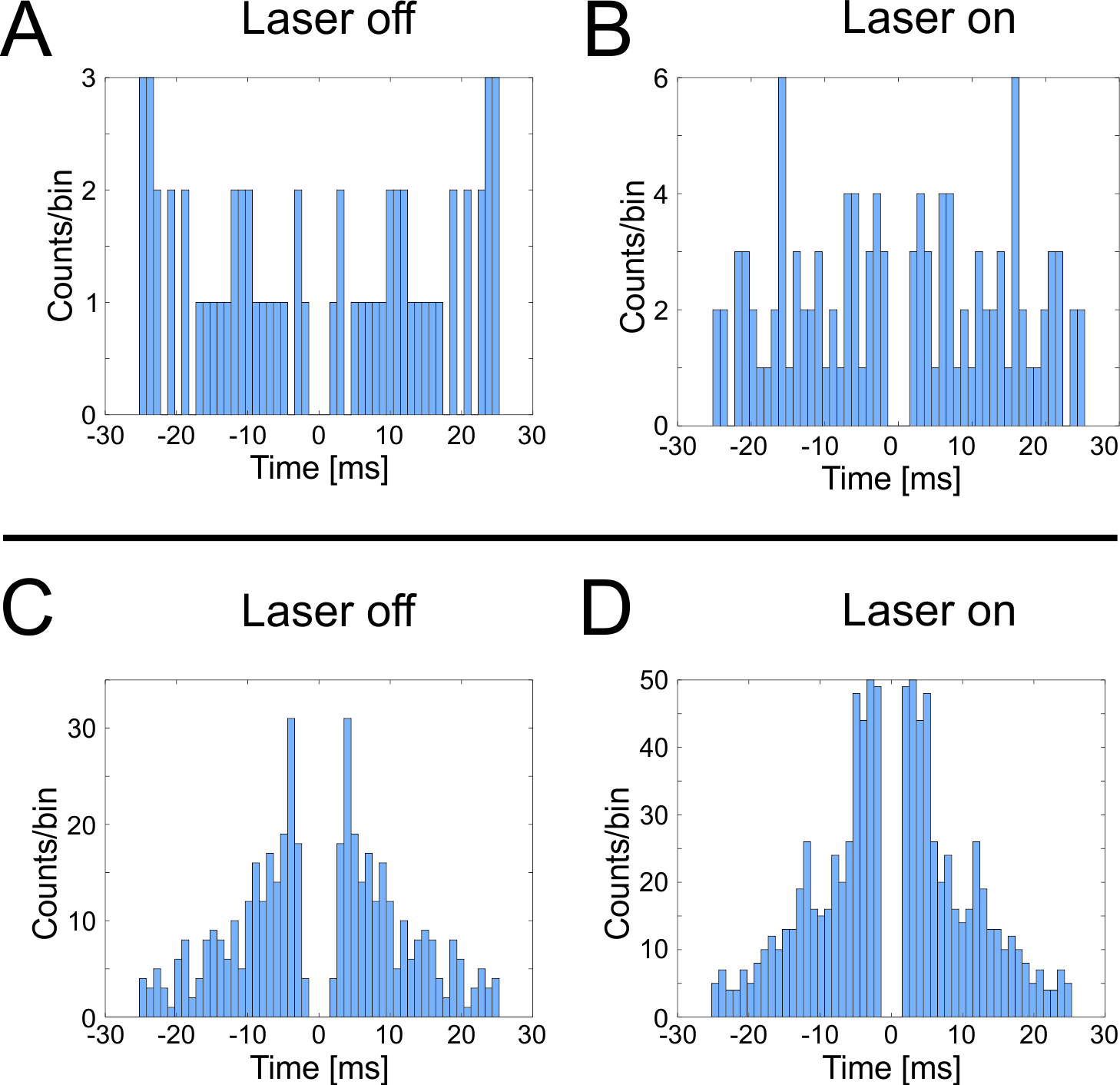

Supplement: S4 Fig — The related A-B and C-D subfigures present the first laser off (A and C) and the laser on (B and D) conditions. (TIF) [file pone.0221510.s004.tif]

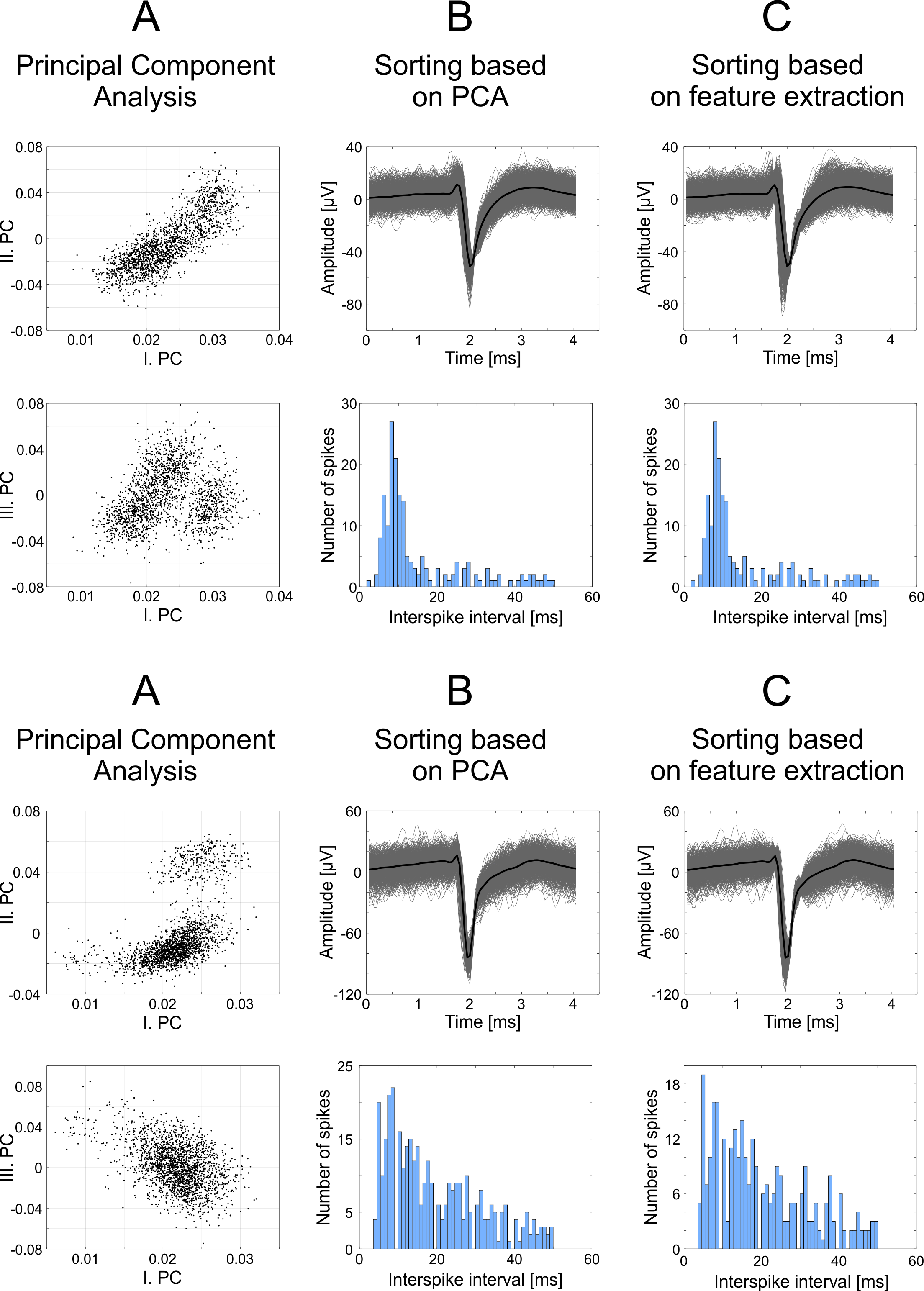

Supplement: S5 Fig — The Principal Component Analysis of the laser noise free, band-pass filtered data (A) in the two cases of the presented single unit activities of Fig 8. The detected and sorted SUA waveforms with their averages based on the PCA (B, top) and based on the feature extraction methods (C, top). The interspike interval (ISI) violators based on the PCA (B, bottom) and based on the feature extraction methods (C, bottom). (TIF) [file pone.0221510.s005.tif]
